# A systematic review of EEG based automated schizophrenia classification through machine learning and deep learning

**DOI:** 10.3389/fnhum.2024.1347082

**Published:** 2024-02-14

**Authors:** Jagdeep Rahul, Diksha Sharma, Lakhan Dev Sharma, Umakanta Nanda, Achintya Kumar Sarkar

**Affiliations:** ^1^Department of Electronics and Communication Engineering, Rajiv Gandhi University, Arunachal Pradesh, India; ^2^Department of Electronics and Communication, Indian Institute of Information Technology, Sri City, India; ^3^School of Electronics Engineering, VIT-AP University, Amrawati, India

**Keywords:** EEG, Schizophrenia (SCZ), AI, machine learning, deep learning, classification

## Abstract

The electroencephalogram (EEG) serves as an essential tool in exploring brain activity and holds particular importance in the field of mental health research. This review paper examines the application of artificial intelligence (AI), encompassing machine learning (ML) and deep learning (DL), for classifying schizophrenia (SCZ) through EEG. It includes a thorough literature review that addresses the difficulties, methodologies, and discoveries in this field. ML approaches utilize conventional models like Support Vector Machines and Decision Trees, which are interpretable and effective with smaller data sets. In contrast, DL techniques, which use neural networks such as convolutional neural networks (CNNs) and long short-term memory networks (LSTMs), are more adaptable to intricate EEG patterns but require significant data and computational power. Both ML and DL face challenges concerning data quality and ethical issues. This paper underscores the importance of integrating various techniques to enhance schizophrenia diagnosis and highlights AI’s potential role in this process. It also acknowledges the necessity for collaborative and ethically informed approaches in the automated classification of SCZ using AI.

## 1 Introduction

**FIGURE 1:**
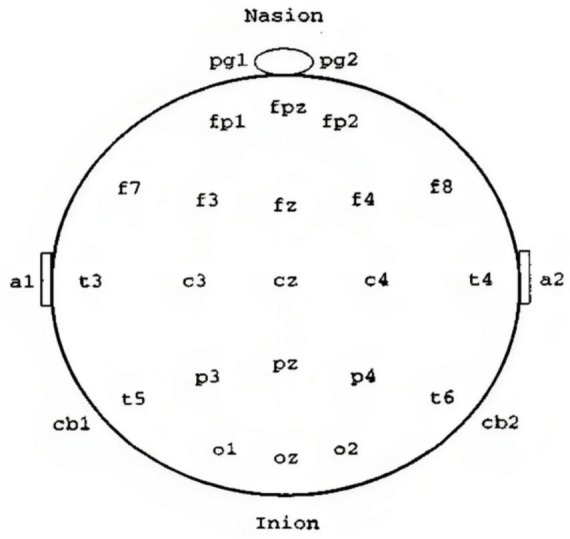
The 10–20 system guides EEG electrode placement (Cooper et al., 2014).

The electroencephalogram (EEG), commonly known as brain waves, is a crucial tool that shows the electrical activity in the brain (Rangayyan, 2015). It facilitates our comprehension of distinct brain regions such as the cerebrum, cerebellum, brain stem, and thalamus. The cerebrum has two hemispheres and a complex outer layer called the cerebral cortex, composed of intricate neuron arrangements. Below the cortex, nerve fibers project and form connections with other brain regions and the peripheral nervous system. The EEG is generated by cortical potentials resulting from interactions among cell bodies and dendrites of pyramidal neurons (Cooper et al., 2014). The scalp acts as a medium for capturing signals from the brain’s intrinsic processes, cognitive activities, and responses to external stimuli, detected by surface electrodes. Scalp EEG provides a consolidated representation of neural activity across various cerebral areas. In medical environments, multiple EEG channels are concurrently recorded from diverse scalp locations to compare activities in various regions (Hughes, 1983; Nahm et al., 1999). The International Federation of Societies for Electroencephalography and Clinical Neurophysiology recommends the 10–20 system for electrode placement (Cooper et al., 2014), ensuring equal spacing for symmetrical positioning and method for placing electrodes during EEG recordings is presented in Figure 1. For specific cases like monitoring sleep or detecting seizures, a more extended recording with multiple channels might be needed. Specialized techniques, like needle electrodes or recording from an exposed part of the cortex, enhance EEG capabilities (Tatum and William, 2021). Different techniques, such as rest or exposure to stimuli like light or sound, help record the EEG in various states (Sarma and Barma, 2009).

Electroencephalogram signals demonstrate distinct rhythmic or periodic patterns, with frequency bands characterized by specific terms. The terminology for EEG frequency bands includes Delta (δ) with a frequency range of 0.5 to 4 Hz, Theta (θ) spanning from 4 to 8 Hz, Alpha (α) ranging between 8 and 13 Hz, and Beta (β) exceeding 13 Hz (Babiloni et al., 2020). Each band represents a different spectrum of electrical activity in the brain, and these classifications are fundamental in EEG analysis, providing insights into various cognitive states and neurological conditions. EEG rhythms intricately reflect a range of physiological and cognitive processes (Miller, 2007). The alpha rhythm, a prevailing resting pattern in the brain, is frequently observed in relaxed adults, particularly in the occipital area with synchronized activity on both sides (Niedermeyer, 1997). During tasks such as listening or mental arithmetic with closed eyes, strong alpha waves emerge, diminishing when the eyes open in response to visual stimuli. As individuals progress through distinct sleep stages, the alpha wave transitions to slower rhythms. Theta waves appear during the initial stages of sleep, while delta waves become prominent in deeper sleep. High-frequency beta waves characterize background activity in individuals experiencing heightened intensity or anxiety. Any deviation from the anticipated rhythm in a specific state may suggest abnormality (Dang-Vu et al., 2008). For instance, the presence of abnormal slow waves like delta or theta during wakefulness is considered atypical. Abnormal slow waves in corresponding regions can be induced by focal brain injuries or tumors. Additionally, a one-sided depression (left-right asymmetry) in rhythm may indicate disruptions in cortical pathways, while the presence of spikes and sharp waves could signal the existence of epileptogenic regions in specific parts of the brain (Yang et al., 2021).

Electroencephalogram can be used to detect and study various mental health conditions, including epilepsy (Cascella et al., 2009), schizophrenia, bipolar disorder, major depressive disorder (Yasin et al., 2021), ADHD (Lenartowicz and Loo, 2014), anxiety disorders (Tolin et al., 2020), sleep disorders (Peter-Derex et al., 2021), neurodevelopmental disorders (Lau-Zhu et al., 2019), traumatic brain injury (Rapp et al., 2015), and dementia (Custodio et al., 2020). It provides insights into brain activity and abnormalities associated with these conditions, assisting in diagnosis and treatment planning. However, EEG is typically used alongside other clinical assessments for a comprehensive evaluation. Expert interpretation by professionals in neurology or psychiatry is crucial. Timely medication and consultations with doctors can be crucial for saving the lives of patients. However, schizophrenia is a serious and chronic mental health disorder that affects a person’s thinking, emotions, and behavior (Aggernaes, 1994). People with schizophrenia often experience a distorted perception of reality, which can include hallucinations (seeing or hearing things that others don’t), delusions (false beliefs), disorganized thinking, and impaired social functioning. The exact cause of schizophrenia is not known, but it is likely to result from a combination of genetic, biological, and environmental factors (Tandon et al., 2008).

The 11*^th^* revision of the International Classification of Diseases (ICD-11), approved by the World Health Assembly, includes a section on “Schizophrenia Spectrum and Other Primary Psychiatric Disorders.” This part of the ICD-11 covers various mental health conditions related to schizophrenia and other primary psychiatric disorders. Schizophrenia is a serious mental illness affecting about 20 million people worldwide (He et al., 2020). Diagnosis is usually based on observed symptoms like hallucinations and disordered speech, along with persistent disengagement from work or social activities. Unlike some physical illnesses, there are no clear biological markers for schizophrenia (Marder and Galderisi, 2017). Brain activity is affected, but other mental illnesses like bipolar disorder or ADHD also influence baseline brain activity (Newson and Thiagarajan, 2019). It’s common for schizophrenia to be confused with other disorders like depression or bipolar disorder, emphasizing the challenge of accurately identifying mental health conditions (Pearlson, 2015). However, a comprehensive diagnosis involves combining EEG findings with clinical assessments, interviews, and possibly other neuroimaging techniques. Trained professionals interpret EEG results within the broader context of an individual’s clinical presentation. Ongoing research aims to enhance our understanding of schizophrenia and improve diagnostic accuracy through neuroimaging methods.

**FIGURE 2:**
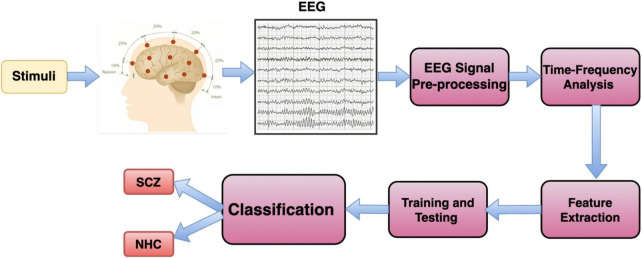
Block diagram for AI-based Schizophrenia classification.

Recently, artificial intelligence (AI) has been used in many areas, including student engagement, virtual reality therapy, text sorting, cybersecurity, detecting and managing diseases, elderly care, analyzing biological data, addressing pandemics, and improving healthcare (Shaffi et al., 2023). In healthcare, AI can help prevent diseases and improve the quality of life. It’s especially useful for accurately diagnosing diseases. AI, particularly machine learning and deep learning, can analyze complex medical data more accurately and quickly, thanks to faster GPUs (graphics processing units) and available datasets (Madiajagan and Sridhar Raj, 2019). A Block diagram for AI-based Schizophrenia classification is illustrated in Figure 2.

AI research in this area involves using both traditional machine learning (ML) and deep learning (DL) methods. The algorithm for diagnosing Schizophrenia (SCZ) using AI includes several steps: preprocessing, feature extraction and selection, and classification. When it comes to diagnosing SCZ through EEG signals, feature extraction is particularly crucial (Sadeghi et al., 2022). In traditional ML, the features extracted from EEG signals are typically divided into four categories: time, frequency, time-frequency, and non-linear fields. These categories help in analyzing different aspects of the EEG signals for a more comprehensive understanding in the diagnosis process. Creating features manually from EEG signals has limitations in uncovering complex characteristics within the data, hindering optimal performance (Khosla et al., 2020). Additionally, selecting effective feature extraction methods for different EEG data structures is challenging, time-consuming, and may not perform well with large datasets, reducing their effectiveness. The deep learning (DL) model demonstrated the capability to manage large datasets, although it required considerably more time for both training and testing in comparison to machine learning (ML) methods (Janiesch et al., 2021).

## 2 Machine learning vs. deep learning for EEG

**FIGURE 3:**
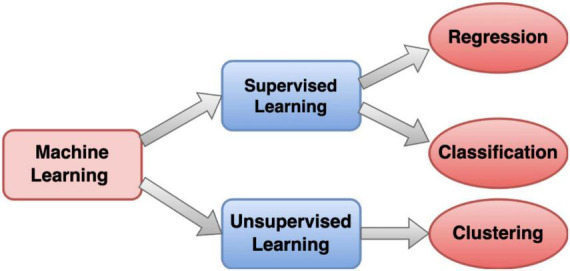
Machine Learning model block diagram representation.

In the realm of EEG analysis using traditional machine learning, a systematic two-step process is employed. Firstly, features are manually extracted from EEG signals, encompassing characteristics like statistical measures or power spectral density. The quality of this feature engineering profoundly impacts the performance of subsequent machine learning models. Common algorithms utilized for EEG classification include Support Vector Machines (SVM), Decision Trees (DT), Random Forests (RF), Logistic Regression (LR), k-Nearest Neighbor (kNN), Naïve Bayes (NB), Boosted tree (BT), Adaboost and other related algorithms (Chen et al., 2014; Rahul et al., 2021; de Miras et al., 2023; Kumar et al., 2023). A pictorial form of machine learning categorization is shown in Figure 3. One notable advantage lies in the interpretability of these models, as the explicitly defined features allow users to comprehend the factors influencing the model’s decisions. Additionally, traditional machine learning models may demonstrate robustness with smaller datasets, making them suitable for scenarios where data availability is limited (Qayyum et al., 2021; Baygin et al., 2023).

**FIGURE 4:**
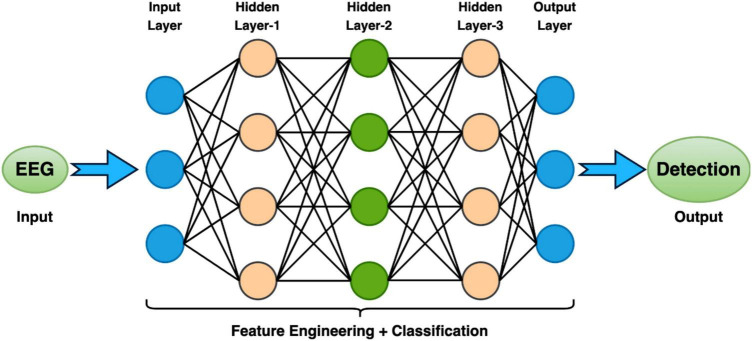
A Deep Learning model consists of an input layer, hidden layers, and an output layer.

**FIGURE 5:**
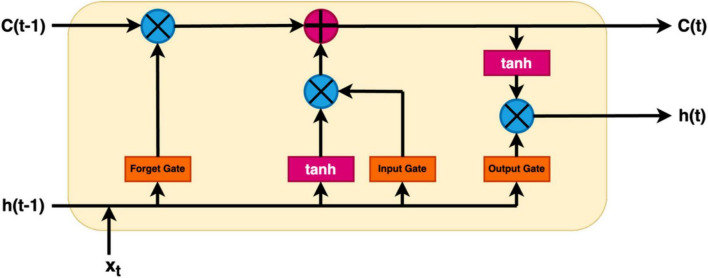
A module of LSTM network.

In the context of EEG analysis, deep learning adopts a distinctive approach by training artificial neural networks to directly glean hierarchical representations from raw EEG data. This obviates the need for manual feature extraction, enabling the model to autonomously discern intricate patterns. Notably, Convolutional Neural Networks (CNNs), Deep Neural Network (DNN), Transfer Learning Models (TLM), Deep Belief Network (DBM), Recurrent Neural Networks (RNNs), Long Short-Term Memory (LSTM), Bi-directional-LSTM (Bi-LSTM) and others related model within the deep learning paradigm excel in learning spatial and temporal dependencies from raw EEG signals, making them adept at tasks like image classification and sequential data analysis, respectively (Tan et al., 2017; Merlin Praveena et al., 2022; Rahul and Sharma, 2022; Chaitanya et al., 2023). However, deep learning models often necessitate substantial datasets for effective generalization, and data augmentation techniques may be required to address data scarcity. While these models can achieve high performance, they are often perceived as “black boxes” due to the complexity of the learned representations, posing challenges in understanding their internal workings. Furthermore, the computational demands for training deep learning models, particularly large neural networks, are significant, requiring powerful GPUs. Choosing between deep learning and machine learning for EEG analysis involves careful consideration of several factors. Deep learning is advantageous for handling raw and complex EEG data, where automatic feature learning is beneficial, while machine learning may suffice for datasets with well-defined features (Li et al., 2020). Computational resources play a crucial role, as deep learning, especially with large neural networks, demands substantial computing power. If interpretability is paramount, as in medical applications, machine learning’s transparent nature may be preferred over the perceived nature of deep learning models. The specific EEG analysis task, whether it involves classification, segmentation, or anomaly detection, also influences the choice of methodology. Ultimately, practitioners weigh these factors to determine the most suitable approach based on their data characteristics, available resources, interpretability needs, and analysis objectives. A Deep Learning model consists of an input layer, hidden layers, and an output layer is demonstrated in Figure 4. Additionally, a module of LSTM network is shown in Figure 5.

## 3 Literature search

**FIGURE 6:**
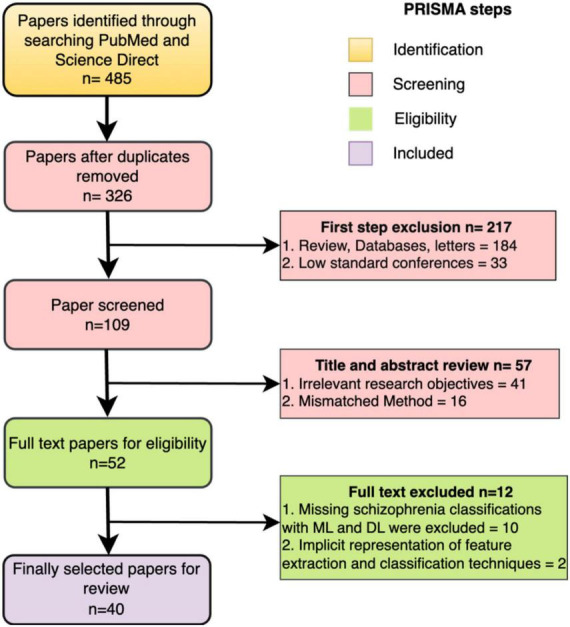
PRISMA flowchart of literature search procedures.

**FIGURE 7:**
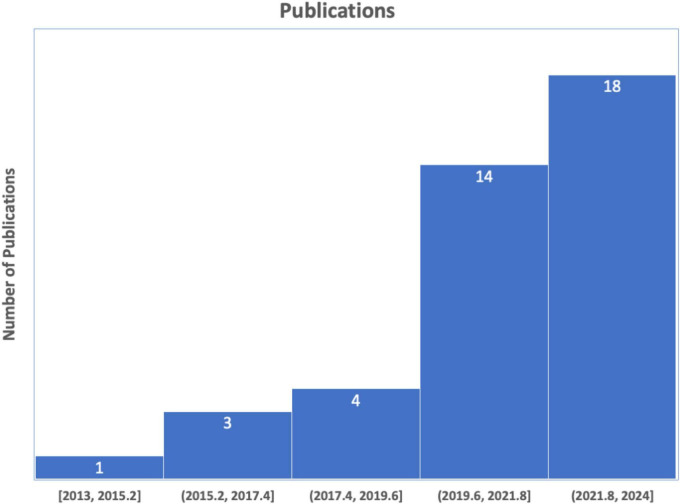
The total number of papers on schizophrenia classification.

We conducted a comprehensive literature search following the PRISMA (Preferred Reporting Items for Systematic Reviews and Meta-Analyses) (Moher et al., 2009), as illustrated in Figure 6. This search specifically focused on the study concerning the classification of schizophrenia using EEG. We began by carefully articulating the study issue of schizophrenia to generate a targeted route for our investigation. We compiled an exhaustive list of keywords and search terms linked to schizophrenia classification using EEG, including variations to ensure inclusivity. In the initial phase of the identification process, PubMed and Science Direct were searched using the terms “Automatic detection of Schizophrenia,” “Automatic classification of Schizophrenia,” “Automatic classification of Schizophrenia using Machine learning,” and “Automatic classification of Schizophrenia using Deep learning,” covering the years from 2013 to November 2023, yielding a total of 485 articles. The subsequent screening phase involved excluding items not in English, as well as reviews, databases, or letters. Further screening based on each article’s title and abstract identified 57 with unrelated study objectives and unmatched techniques, leaving 52 items for thorough inspection. Additionally, 10 publications were excluded for not containing schizophrenia classifications with ML and DL, and 2 studies lacked clear representations of feature extraction methods or classification algorithms. After identifying, reviewing, and verifying that 40 papers met the inclusion criteria, the procedure was successfully concluded. The studies reviewed were distributed based on their year of publication. The databases utilized in the 40 studies are outlined in Figure 7.

## 4 Schizophrenia classification using machine learning

Detecting schizophrenia using machine learning (ML) is a promising area of research with significant potential for improving early diagnosis and intervention. ML techniques can analyze various data sources, including biomedical signals, to identify patterns and markers associated with schizophrenia. In this study, the details of papers reviewed are summarized in Table 1, where out of 40 papers, 24 specifically addressed Schizophrenia classification using machine learning.

**TABLE 1:** Studies employing Machine Learning methods for detection of SCZ.

Study	Preprocessing	Method	Signal	Database	Accuracy
Vázquez et al., 2021	Low- and high-pass Butterworth filters	RF Classifier	EEG	IPN (Olejarczyk and Jernajczyk, 2017)	NR
Rajesh and Sunil Kumar, 2021	Symmetrically Weighted local binary patterns (SLBP) and correlation	Logit Boost classifier	EEG	Laboratory for Neurophysiology and Neuro-Computer Interfaces, MHRC	91.66
Agarwal and Singhal, 2023	Fast Fourier transform (FFT) and statistical feature	SVM, KNN, BT, and DT	EEG	IPN (Olejarczyk and Jernajczyk, 2017) and Kaggle SCZ dataset (Ford et al., 2014)	99.25
Khare and Bajaj, 2022	Robust variational mode decomposition (RVMD)	Optimized extreme machine classifier	EEG	Kaggle SCZ dataset (Ford et al., 2014)	92.93
Aydemir et al., 2022	CGP17Pat and Iterative neighborhood component analysis (INCA)	KNN	EEG	IPN (Olejarczyk and Jernajczyk, 2017)	99.91
Jahmunah et al., 2019	Butterworth filter and Segmentation	DT, Linear-Discriminant Analysis (LDA), KNN, Probabilistic-Neural- Network (PNN), and SVM	EEG	IPN (Olejarczyk and Jernajczyk, 2017)	92.91
Devia et al., 2019	Butterworth filter and Independent component analysis (ICA)	LDA, and Rule-based classifier	EEG	Private	71
Neuhaus et al., 2013	Digital filters and ICA	KNN, LDA, SVM,	EEG	Private	72.4
Luján et al., 2022	Spatial filters and Bandpass filter	SVM, Bayesian LDA, Gaussian NB, KNN, Adaboost, and Radial basis function (RBF)	EEG	Private	93.40
Khare and Bajaj, 2021	Flexible tunable Q wavelet transform (F-TQWT)	Flexible least square support vector machine (F-LSSVM) classifier and grey wolf optimization (GWO) algorithm	EEG	Kaggle SCZ dataset (Ford et al., 2014)	91.39
Zandbagleh et al., 2022	EEGLAB and ICA	KNN, LDA, and SVM	EEG	Private	89.21
Du et al., 2020	Wavelet Transform	Non-linear dynamic and Functional brain networks	EEG	Private	76.77
Aksöz et al., 2022	Finite impulse response (FIR) filter	KNN, ANN, and SVM	EEG	Kaggle SCZ dataset (Ford et al., 2014)	93.9
Najafzadeh et al., 2021	Butterworth filter	Adaptive neuro fuzzy inference system (ANFIS), SVM, and ANN	EEG	IPN (Olejarczyk and Jernajczyk, 2017)	100
Azizi et al., 2021	Bandpass filter and ICA	LR classifier	EEG	IPN (Olejarczyk and Jernajczyk, 2017)	97
Shim et al., 2016	Bandpass filter	SVM	EEG	Private	88.24
Kim et al., 2021	Bandpass filter	SVN	EEG	IPN (Olejarczyk and Jernajczyk, 2017)	76.85
Keihani et al., 2022	Bandpass filter	SVM and Bayesian optimization	EEG	IPN (Olejarczyk and Jernajczyk, 2017)	90.93
Prabhakar et al., 2020	ICA	Black Hole (BH) optimization and SVM	EEG	IPN (Olejarczyk and Jernajczyk, 2017)	92.17
Kim et al., 2020	Bandpass filter and notch filter	LDA	EEG	Private	88.10
Jeong et al., 2017	Bandpass filter and ICA	LDA	EEG	Private	98
Lai et al., 2019	Bandpass filter and ICA	NB and SVM	EEG	Private	86.3
Guo et al., 2022	Vietoris–Rips filtering algorithm	Bottleneck and Wasserstein distances	EEG	Private	NR
Santos-Mayo et al., 2016	Bandpass filter + ICA + Grand average + Segmentation	Multilayer Perceptron (MLP) and SVM	EEG-ERP	Private	93.42

IPN, Institute of Psychiatry and Neurology; MHRC, Mental Health Research Center; ERP, Evoked Related Potential.

The Table 1 presents a diverse array of methodologies applied in EEG signal analysis, offering insights into various preprocessing techniques and classification methods. Notably, reference (Agarwal and Singhal, 2023) employs a robust strategy that integrates FFT and statistical features with SVM, KNN, BT, and DT classifiers, achieving an impressive accuracy of 99.25% across both the IPN and Kaggle SCZ datasets. Conversely, reference (Devia et al., 2019) reports a lower accuracy of 71% on private EEG dataset, attributed to the use of a Butterworth filter and ICA in conjunction with a small dataset. The integration of spatial filters and a bandpass filter in reference (Luján et al., 2022), utilizing six different classifiers where RBF stands out, results in a notable accuracy of 93.40% on private data. The method described in Khare and Bajaj (2022), which uses RVMD for preprocessing and optimized extreme machine learning on the Kaggle SCZ dataset, achieved lower accuracy but outperformed (Devia et al., 2019). The approach proposed in Aydemir et al. (2022), employing CGP17Pat for feature extraction and KNN for classification, obtained a very high accuracy of 99.91%, though it did not surpass the results achieved by Najafzadeh et al. (2021). These studies employ diverse databases, including IPN, Kaggle SCZ, and private datasets, showcasing the adaptability of methods across various contexts. Various preprocessing techniques, such as wavelet transform (Du et al., 2020), FIR filter (Aksöz et al., 2022), and adaptive neuro-fuzzy inference system (ANFIS) (Najafzadeh et al., 2021), highlight the richness and diversity of approaches in EEG signal analysis. Noteworthy is the study in Najafzadeh et al. (2021), which achieves a perfect accuracy of 100% using a Butterworth filter alongside ANFIS, SVM, and ANN. The methods in Shim et al. (2016); (Jeong et al., 2017; Lai et al., 2019; Kim et al., 2020, 2021; Azizi et al., 2021; Keihani et al., 2022), and (Santos-Mayo et al., 2016), which use a bandpass filter in combination with other preprocessing techniques, fail to achieve the impressive accuracy seen in Aydemir et al. (2022); (Agarwal and Singhal, 2023), and (Najafzadeh et al., 2021). The studies in Vázquez et al. (2021) and (Guo et al., 2022), employing Butterworth and Vietoris–Rips filtering for preprocessing and RF classifier and Bottleneck and Wasserstein distances, respectively, for classification of SCZ, did not report their results in terms of accuracy. The methods in Jahmunah et al. (2019); (Khare and Bajaj, 2021; Rajesh and Sunil Kumar, 2021; Luján et al., 2022; Zandbagleh et al., 2022), and (Aksöz et al., 2022) achieved moderate accuracy with different preprocessing techniques. However, the approaches in Neuhaus et al. (2013) and (Du et al., 2020) secure lower accuracies but still surpass the performance of Devia et al. (2019). The reported accuracies highlight the significant role of preprocessing and classification choices in EEG signal processing effectiveness. These studies underscore the need for context-specific, tailored approaches and the importance of balancing preprocessing and classification methods for optimal results.

## 5 Schizophrenia classification using deep learning

The deployment of these sophisticated neural networks enables the creation of a dynamic and adaptive system that can discern subtle and complex patterns indicative of schizophrenia. This process is vital in enhancing the accuracy of detection, as it allows the model to uncover intricate relationships and features that might be challenging for traditional methods to identify. The amalgamation of neural network capabilities with deep learning principles positions these models as powerful tools in the quest for more effective and nuanced approaches to schizophrenia detection. In this section, the details of the papers reviewed are summarized in Table 2, where out of 40 papers, 16 specifically addressed schizophrenia classification using deep learning.

**TABLE 2:** Studies using Deep Learning methods for detection of SCZ.

Study	Pre-processing	Method	Signal	Database	Accuracy (%)
Bagherzadeh et al., 2022	Butterworth filters and Transfer Entropy (TE)	VGG-16, ResNet50V2, InceptionV3, EfficientNetB0, DenseNet121, and CNN-LSTM	EEG	IPN (Olejarczyk and Jernajczyk, 2017)	99.90
Aslan and Akin, 2022	Continuous Wavelet Transform	Visual Geometry Group-16 (VGG16), an Advanced convolutional neural network (CNN)	EEG	1. Private 2. IPN (Olejarczyk and Jernajczyk, 2017)	99.5
Supakar et al., 2022	Dimensionality reduction algorithm	RNN-LSTM	EEG	Laboratory for Neurophysiology and Neuro-Computer Interfaces (Shishkin et al., 2011)	98
Shalbaf et al., 2020	Wavelet Transform	AlexNet, ResNet-18, VGG-19, Inception-v3, and SVM	EEG	IPN (Olejarczyk and Jernajczyk, 2017)	98.60
Soria Bretones et al., 2023	Digital filters and Fuzzy means clustering	Artificial neural network based on RBF	EEG	Psychiatry Department of Virgen de la Luz Hospital in Cuenca	93
(Siuly et al., 2023)	Average filtering	Deep ResNets, softmax layer and deep features with SVM	EEG	Kaggle SCZ dataset (Ford et al., 2014)	99.23
Siuly et al., 2022	Average filtering	GoogleNet and deep features, SVM	EEG	Kaggle SCZ dataset (Ford et al., 2014)	98.84
Shoeibi et al., 2021	Segmentation, Denoising, Normalization	1-D-CNN, LSTM, and 1-D-CNN + LSTM	EEG	IPN (Olejarczyk and Jernajczyk, 2017)	99.25
Phang et al., 2019	Connectivity measures (VAR coefficients and PDCs)	Multi domain-CNN	EEG	EEG SCZ (Gorbachevskaya and Borisov, 2002)	91.69
Singh et al., 2021	Bandpass filter, segmentation, and Fast Fourier transform (FFT)	CNN + LSTM	EEG	1. EEG SCZ (Gorbachevskaya and Borisov, 2002) 2. IPN (Olejarczyk and Jernajczyk, 2017)	98.56
Khare et al., 2021	Short Term FFT, Continuous WT, and SPWVD	AlexNet, ResNet50, VGG16, and CNN	EEG	Kaggle SCZ dataset (Ford et al., 2014)	93.36
(Sun et al., 2021)	Fourier transform (FT)	CNN + LSTM	EEG	Huilongguan Hospital	99.22
Supakar et al., 2022	Random projection	RNN + LSTM	EEG	Laboratory for Neurophysiology and Neuro-Computer Interfaces (Shishkin et al., 2011)	98
Hassan et al., 2023	Digital bandpass filter	Hybrid Classifier (CNN + ML)	EEG	IPN (Olejarczyk and Jernajczyk, 2017)	98.05
Bao et al., 2023	Notch filter + Principal component analysis (PCA)	CNN and temporal convolution networks (TCNs)	EEG	IPN (Olejarczyk and Jernajczyk, 2017)	99.57
Shen et al., 2023	Continuous wavelet transform (CWT) + Digital bandpass filter	Customized 3D-CNN	EEG	Laboratory for Neurophysiology and Neuro-Computer Interfaces (Shishkin et al., 2011)	98.89

Table 2 provides a comprehensive summary of various studies that have used deep learning methods for the detection of SCZ using EEG. The method presented in Bagherzadeh et al. (2022) employ Butterworth filters and Transfer Entropy (TE) in conjunction with various deep learning architectures, including VGG-16, ResNet50V2, InceptionV3, EfficientNetB0, DenseNet121, and CNN-LSTM, achieving an impressive accuracy of 99.90% on EEG signals from the IPN database. Aslan and Akin (2022) employs CWT and advanced CNN and VGG16, achieving a high accuracy of 99.5% on both private and IPN EEG data. Supakar et al. (2022) employ a dimensionality reduction algorithm in conjunction with RNN-LSTM, achieving an accuracy of 98% on Laboratory for Neurophysiology and Neuro-Computer Interfaces dataset. Shalbaf et al. (2020) utilize Wavelet Transform with AlexNet, ResNet-18, VGG-19, Inception-v3, and SVM, achieving an accuracy of 98.60% on the IPN dataset. Soria Bretones et al. (2023) introduce digital filters and fuzzy means clustering combined with an artificial neural network based on radial basis function (RBF), achieving an accuracy of 93% on EEG data from the Psychiatry Department of Virgen de la Luz Hospital in Cuenca. Siuly et al. (2023) and (Siuly et al., 2022) apply average filtering with different deep learning architectures, such as deep ResNets, GoogleNet, and SVM, achieving high accuracies of 99.23% and 98.84%, respectively, on the Kaggle SCZ dataset. Shoeibi et al. (2021) employ segmentation, denoising, and normalization techniques with 1-D-CNN, LSTM, and 1-D-CNN + LSTM, achieving an accuracy of 99.25% on EEG signals from the IPN database. Phang et al. (2019) utilize connectivity measures, specifically VAR coefficients and PDCs, with a multi-domain CNN, achieving an accuracy of 91.69% on EEG SCZ data. Singh et al. (2021) combine bandpass filtering, segmentation, and FFT with CNN + LSTM, achieving an accuracy of 98.56% on EEG data from both EEG SCZ and IPN databases. Lastly (Khare et al., 2021) apply STFT, CWT, and SPWVD with various CNN architectures (AlexNet, ResNet50, VGG16), achieving an accuracy of 93.36% on the Kaggle SCZ dataset. Sun et al. (2021) achieved 99.22% accuracy using Fourier Transform with CNN + LSTM, while (Supakar et al., 2022) reported 98% with Random Projection and RNN-LSTM. Hassan et al. (2023) combined a Digital Bandpass Filter with a CNN + ML hybrid, reaching 98.05% accuracy. Notably, (Bao et al., 2023) attained the highest accuracy of 99.57% using Notch Filter, PCA, CNN, and TCNs. Shen et al. (2023) employed Continuous Wavelet Transform and a 3D-CNN, achieving 98.89%. The highest accuracy was reported by Bagherzadeh et al. (2022), achieving an impressive 99.90% and the other hand, the lowest accuracy was noted in the study (Phang et al., 2019), with an accuracy of 91.69%. These varied accuracies highlight the influence of specific preprocessing techniques and neural network architectures on the effectiveness of detecting schizophrenia in EEG.

## 6 EEG datasets for schizophrenia detection

Electroencephalogram signals can be readily obtained from high-quality open datasets accessible to the public. These datasets, which are easily obtainable, open, and shareable, significantly aid independent researchers in developing new algorithms or findings and conducting performance comparisons in studies related to Schizophrenia diagnosis. There are multiple EEG datasets specifically designed for predicting SCZ. However, ethical concerns prevent most of this data from being shared in the public domain, limiting the reproducibility of related works. Some private datasets, including those used in studies (Neuhaus et al., 2013; Santos-Mayo et al., 2016; Shim et al., 2016; Jeong et al., 2017; Devia et al., 2019; Lai et al., 2019; Du et al., 2020; Kim et al., 2020; Aslan and Akin, 2022; Bagherzadeh et al., 2022; Guo et al., 2022; Luján et al., 2022; Zandbagleh et al., 2022) utilized experimental EEG datasets where researchers devised experimental paradigms. Participants provided written informed consent, and approval was obtained from the Institutional Review Board (IRB). The scarcity of publicly available datasets poses a major challenge for researchers exploring diseases and conducting disease prediction and medical pattern detection tasks. Nevertheless, in recent years, several EEG datasets for SCZ diagnosis have become accessible in an open environment, gaining significant attention. In this research domain, there are a few popular publicly available datasets for binary classification of SCZ. The initial dataset is sourced from the Institute of Psychiatry and Neurology in Warsaw, Poland, and is openly accessible in the RepOD dataset (Olejarczyk and Jernajczyk, 2017). The second dataset originates from the Neurophysiology and Neuro-Computer Interfaces laboratory at the Mental Health Research Center (MHRC), Russia, and is publicly available. The third and less-explored SCZ EEG dataset is collected under a project of the National Institute of Mental Health (NIMH; R01MH058262) and is publicly available on the Kaggle platform (Ford et al., 2014).

## 7 Challenges in classification of schizophrenia using ML and DL

Machine learning (ML) faces challenges in classifying schizophrenia due to diverse and limited datasets, hindering the development of generalized models. The complexity of schizophrenia symptoms makes it hard to choose relevant features and interpret model results, affecting our understanding of clinical significance. Imbalanced datasets, ethical concerns, and the need for collaboration between machine learning experts and clinicians further complicate building accurate and ethical classification models. Overcoming these challenges requires improving data quality, fostering collaboration, and addressing ethical considerations to integrate machine learning effectively into clinical practices for schizophrenia diagnosis. Traditional ML distinguishes itself through a multi-stage approach involving pre-processing, feature selection, and extraction. In ML, the need for predefined feature engineering is prevalent, and the models are characterized by task-specific features (Cortes-Briones et al., 2022). This approach may struggle with adaptability to various data types or applications, and the learning process often lacks autonomy. While ML provides a structured and interpretable framework, its reliance on explicit feature engineering can limit its ability to handle complex relationships within the data. Despite these limitations, ML remains a valuable tool in various domains, contributing insights and predictions based on well-defined features and structured algorithms (Bzdok and Meyer-Lindenberg, 2018).

Several challenges are associated with deep learning (DL) models. One significant hurdle is the substantial need for extensive training data to construct effective DL models. Transfer learning, a strategy leveraging data from related tasks, can partially address this issue, improving the model’s performance. However, it does not entirely replace the requirement for original data (Sharma et al., 2023). Another challenge involves dealing with unbalanced data, which is common in biological datasets where negative samples often outnumber positive ones. Training DL models on skewed data may lead to unexpected outcomes, and the impact of imbalanced data on model performance has been extensively studied (Johnson and Khoshgoftaar, 2019). Additionally, uncertainty scaling is crucial in healthcare applications to assess the accuracy of ML and DL-based diagnoses, preventing overconfident predictions. Catastrophic forgetting is another issue, occurring when new information disrupts previously learned knowledge in simple DL models. To mitigate this problem, training a new model from scratch with both old and new data is a recommended solution. DL models also face the risk of overfitting during training due to numerous interrelated parameters, impairing their overall effectiveness. Inadequate training data further contributes to overfitting, causing the learned distribution to deviate from the true distribution. Additionally, the vanishing gradient problem arises in DL, especially during backpropagation, when weights may not update effectively, leading to termination of the neural network training process (Li et al., 2019).

The key highlights of this study are summarized as follows:

1. This study provides a detailed review of DL and ML methodologies applied in the detection of SCZ.
2. This study evaluates the shortcomings of current DL and ML methodologies, proposing possible solutions.
3. This study offers a comprehensive analysis of all relevant parameters from existing studies that use DL and ML techniques, specifically within the comparison and discussion sections.
4. It also explores the future of integrating EEG and neuroimaging data to diagnose SCZ through AI algorithms.

## 8 Conclusion

This paper finds into the application of artificial intelligence, specifically machine learning (ML) and deep learning (DL), in the classification of SCZ using EEG signals. The review highlights that traditional ML models like Support Vector Machines and Decision Trees are interpretable and robust with smaller datasets, while DL methods using neural networks demand more data and computational resources but adapt well to complex EEG patterns. The review encompasses a thorough examination of methodologies, challenges, and findings in this realm. Both approaches face challenges in data quality, interpretability, and ethics. The diverse techniques highlighted in reviewed studies emphasize the need for a balanced, context-specific approach. While AI holds promise for advancing SCZ diagnosis, interdisciplinary collaboration and ethical considerations are crucial for its effective integration into mental health.

## Data availability statement

The original contributions presented in this study are included in this article/supplementary material, further inquiries can be directed to the corresponding author.

## Author contributions

JR: Investigation, Writing−original draft. DS: Data curation, Visualization, Writing−original draft. LS: Conceptualization, Formal Analysis, Supervision, Validation, Writing−review and editing. UN: Funding acquisition, Resources, Supervision, Writing−review and editing. AS: Project administration, Supervision, Writing−review and editing.
